# Selections and Abstracts

**Published:** 1900-02

**Authors:** 


### SELECTIONS AND ABSTRACTS.

---

### THE NATURE AND TREATMENT OF ACUTE INFECTIONS OF THE GENITAL TRACT IN WOMEN.

BY J. L. ROTHROCK, M.D., St. Paul, Minn.

The great frequency and often the disastrous consequences of inflammatory disease of the organs of generation in women make the subject of acute infection of the genital tract one of universal interest and importance. The exhaustive researches of recent years, notably by Bumm, Winter, Doederlein, Menge and Kroenig, Williams and others, with especial reference to puerperal infection, have elucidated many hitherto obscure points concerning the etiology of infections in the non-puerperal woman. It may be said that almost without exception infection gains entrance to the genital tract from without. The one possible exception is infection through the blood, which is so rare as not to deserve mention.

It is not at all difficult to understand how infection may gain entrance to the genital tract. In the married woman the repeated acts of coitus constantly favor not only the introduction of infectious micro-orangisms, but frequently, no doubt, create the atrium of entrance—which, for the usual bacteria of wound infection, as we shall point out later, is such an essential condition.

The exhaustive researches of Menge have shown that while the skin in close proximity to the vulva normally contains the usual bacterial flora of the skin elsewhere, the mucous membrane of the vulva seldom contains pathogenic bacteria. Thus from an examination of 70 cases, three times he found staphylococcus pyogenes aureus, twice streptococcus pyogenes, and twice the colon bacillus. He attributes this comparative immunity to the germicidal power of the vaginal secretion.

Of all forms of acute infection of the vulva, gonorrheal infection is by far the most frequent, constituting about three-fourths of all cases. Next comes staphylococcus infection, which usually produces a pustular inflammation resembling furunculosis of the skin. Less frequently we may have an erysipelatous inflammation due to the streptococcus.

Gonorrheal infection may involve the entire vulva, or be confined to the peri-urethral or vulvo-vaginal glands. In childhood the vulva is the most common seat of gonorrheal infection, while in adults we frequently see the trouble localized in the urethra, peri-urethral or vulvo-vaginal glands. The bacterial flora of the vagina in health has been the subject of much controversy. The researches of Doederlein and Menge, however, render it certain that while in health the vagina may contain a variety of bacteria, they are usually non-pathogenic. In fact, Menge even went so far as to introduce into the vagina cultures of pathogenic bacteria. For example, he introduced the bacillus pyocyanus 23 times, the staphylococcus pyogenes aureus 30 times and the streptococcus pyogenes 27 times, and after varying periods of time found them to have entirely disappeared without causing any disturbance. Only once did he succeed in exciting a vaginitis, and that was by the staphylococcus pyogenes aureus, which was introduced into a not altogether healthy vagina.

The author remarks that he has no doubt that the streptococcus may also, under favorable conditions, produce a vaginitis. All these bacteria were cultivated from sources which would guarantee their virulence; the bacillus pyocyanus, for example, from blue pus, the staphylococcus pyogenes aureus from a mammary abscess, while the streptococcus was taken from the lochia of a woman suffering from puerperal infection. The gonococcus occasionally produces inflammation of the vagina, especially in children. In adults with gonorrheal infection the vagina usually escapes. This peculiarity, no doubt, owes its existence to two factors; first, the germicidal power of the vaginal secretion, and second, the character of the epithelial lining of the vaginal mucous membrane.

While the cervical canal contains bacteria, according to Winter, they never are found in health inside the internal os. In support of this view the sterility of the uterine cavity has been repeatedly confirmed by other observers. Here, as elsewhere, the usual bacteria of wound infection may cause inflammation, provided the requisite conditions are present. The mere presence of the given micro-organisms, however, will not necessarily produce inflammation in the absence of an atrium of infection, which is usually to be found in wounds or abrasions, or a lowered vitality of the cells of the organism. Retained secretions or secundines, or the presence of necrotic new growths in the uterine cavity, all favor infection by furnishing a favorable culture media. For this reason infection by pyogenic bacteria is most commonly observed following abortion or labor, though infection may take place during menstruation, in which case the menstrual flow will be suppressed. Suppressed menstruation, therefore, so often attributed to "taking cold," is to be regarded as the result of infection, and on that account is to be viewed with apprehension. Sudden chilling of the surface of the body may, however, predispose to infection by producing internal congestion, thereby lowering the resistance of the organism against infectious bacteria, which may be present.

The cervical canal is a favorite point of attack for the gonococcus, and constitutes one of the chief points of localization of gonorrheal infection in women. Indeed the gonococcus is by far the most common etiological factor concerned in acute infections of the uterine cavity in non-puerperal women. The great tendency of gonorrheal infection to extend by direct continuity to the fallopian tube has been so repeatedly observed by different investigators, that it constitutes the most important form of infection with which we have to deal.

The purpose of this paper is to call attention to the necessity of more exact methods of dealing with acute infections of the genital tract in women. An experience of several years, during which time many cases have come under observation, has convinced the writer that far too little attention is paid to detail in the management of acute infections, and that many conditions subsequently demanding major operations might be prevented if properly treated in the beginning.

The enthusiasm for operative work has, for the moment, overshadowed the less conspicuous, but in no wise less important prophylactic treatment which is justly due our patients. Indeed, so pessimistic a view is taken of gonorrheal infection of the uterus, that we find writers in certain text-books advocating hysterectomy as the most satisfactory method of dealing with the diseased organ. A wide experience in the management of these cases has convinced the writer that this view is as fallacious as would be the amputation of a limb in case of an infected wound, without having first used means to control the infection.

Vulvitis presents little difficulty in treatment. The field of infection is easily accessible, and, except for the more concealed recesses of the urethral and vulvo-vaginal glands, may be easily reached and the infection be speedily brought under control. Absolute cleanliness and a liberal use of some antiseptic in solution of which bichloride of mercury, owing to its inhibitory action against the growth of bacteria, is perhaps the best, should be employed. On the vulva it may be used in a strength of 1:2000 after which the surface should be dried and some dusting powder applied such as aristol or iodol, after which the vulva should be kept separated by a small piece of lint gently tucked between the labiae. In gonorrheal infection this may be supplemented by painting the surface after irrigation with bichloride with a solution of nitrate of silver in the strength of ten grains to the ounce. The vulvo-vaginal and peri-urethral glands require special attention. The infection is confined to the ducts and, owing to their small size, is not easily accessible.

In many instances it is best to at once lay them open so that local application may be brought in contact with every portion of infected lining, after which the process speedily yields to treatment. If the duct is very patulous, a slender probe wrapped with cotton may be easily introduced and after irrigating with a syringe an application may be made, for which purpose a rather strong solution of nitrate of silver (20 grs. to the ounce) or a 2 per cent. solution of formalin have, in the writer's hands, proved efficacious.

Vaginitis, like vulvitis, is easily accessible and correspondingly amenable to treatment. Antiseptic irrigations, preferably by bichloride of mercury in 1:3000 solution should be used, after which the surface should be dried and a dusting powder of aristol or iodoform applied. The walls of the vagina should be kept separated by iodoform or other antiseptic gauze for the purpose of keeping it dry and thus preventing maceration of the partially destroyed epithelial cells. The usual objection to the odor of iodoform is eliminated when used in the vagina, and the dressing may remain longer without becoming foul. In case of both vulvitis and vaginitis the dressing should be made daily and in most instances a few treatments will suffice to effect a cure.

Infection of the uterine cavity offers many more obstacles to rational treatment. On that account two methods have been in common use. On the one hand the ultra-conservative method, consisting in giving antiseptic vaginal douches and leaving the infection to run its course, while on the other hand the radical procedure of curettement has been advised and practiced. The latter as applied to acute infections I mention only to condemn. While not having practiced it myself, I can, nevertheless, speak from observation, since a number of cases have come under my care requiring salpingectomy, with a distinct history of an acute exacerbation immediately following such treatment and to which they could positively date their trouble.

There is no reason why local treatment cannot be rationally carried out in infection of the uterine cavity just as elsewhere, but modern text-books have so discouraged all form of local treatment as applied to gynecological affections, that this practice, once so popular, has largely gone out of fashion.

In this the pendulum of opinion has swung too far and is bound to right itself. In the puerperal woman, or woman who has recently aborted, intra-uterine treatment offers little difficulty, inasmuch as the os is still patulous so that access may be had to the uterine cavity. It is quite the contrary with the non-puerperal, and especially the nulliparous woman, who frequently has a very small os uteri, which not only prevents the free outflow of secretions from the uterus, but likewise renders practically impossible all efforts at intra-uterine treatment. In such cases it has been my custom to proceed as follows: After having prepared the field by careful sterilization, a short sea-tangle tent is introduced, just long enough to reach through the internal os, and which is held in place by a cotton tampon. The smallest size is large enough, and they may be sterilized by dry heat, after which they are kept in ether saturated with iodoform or 5 per cent. carbolic acid in absolute alcohol. The dilatation by these tents is gradual and ordinarily the pain occasioned is slight. The tent may be introduced in the office, after which the patient is directed to go home, and go to bed, and remain there until the tent is removed. The tent is allowed to remain from six to eight hours, at the expiration of which dilatation will be found to be sufficient. The removal of the tent is followed by antiseptic douches. The uterine cavity is now accessible and the secretion from the inflamed membrane may be removed by irrigation. In case there is much mucous present it is best to first use a soda solution or peroxide of hydrogen, after which I prefer bichloride of mercury in solution of the strength of 1:4000. For this purpose I have modified the ordinary Fritch-Bozeman intra-uterine catheter, supplementing it by a fluted cylinder with openings so arranged that the return current escapes along the depressions of the fluted portion, thus bringing it in contact with every portion of the inflamed mucous membrane. After irrigation, applications may be made by means of cotton wrapped on an applicator. In case of gonorrheal infection quite strong applications are necessary in order to reach the gonococci, which are protected by having insinuated themselves between the epithelial cells. For this purpose nitrate of silver in the strength of 40 grains to the ounce, formalin in 2 per cent. solution and 25 per cent. ichthyol in glycerine are useful. The treatment should be made daily, always with the same care as regards antiseptic precautions. For obvious reasons sexual intercourse must be absolutely interdicted during the progress of treatment. Not only does the injury wrought by repeated congestions occasioned by the sexual act prove harmful, but the likelihood of reinfection must be taken seriously into account, especially in cases of gonorrheal infection. Indeed, in many instances it is wise to insist upon the husband being put upon proper treatment for the relief of the chronic infection, which is usually to be found seated in the deeper portions of the uretha. Otherwise this may prove a constant source of reinfection, and in spite of all treatment indefinitely prolong the course of the disease. After a large experience the writer can heartily recommend this mode of treatment. As proof of its value, in no case in which it has been tried has subsequent operation for tubal disease been necessary, and in but five per cent. in which the tubes had not already been involved at the time treatment was begun, was there subsequent extension of the infection to the tubes.—*Northwestern Lancet.*

---

### THE MORTALITY FROM TYPHOID FEVER IN THE PRINCIPAL CITIES OF THE UNITED STATES.

Typhoid fever is generally recognized as coming under the head of preventable diseases, and at no very distant period of time in the future the right to place it in this category of diseases will doubtless be proven. Coming generations will wonder at the sanitary negligence which made possible the annual slaughter of thousands of citizens, most of them at the dawn of their period of greatest activity, that characterized the latter half the nineteenth century.

In the *Medical Record* of August 12th of the past year appeared an article by F. S. Crum on the mortality of typhoid fever in twenty-four American cities, for ten year, from 1889 to 1898 inclusive. The total number of deaths during the ten years above indicated, arranged according to population, was as follows: "New York, 3,533; Chicago, 8,450; Philadelphia, 5,186; Brooklyn, 1,803; St. Louis, 1,712; Boston, 1,604; Baltimore, 1,966; San Francisco, 1,151; Cincinnati, 1,448; Cleveland, 1,663; Buffalo, 1,036; New Orleans, 844; Pittsburg, 2,272; Washington, 1,840; Milwaukee, 625; Newark, 783; Minneapolis, 962; Jersey City, 1,103; Louisville, 1,280; Omaha, 295; Rochester, 400; St. Paul, 489; Providence, 480; Denver, 1,006."

This mortality represents the death-rate per hundred thousand of: New York, 21; Chicago, 65; Philadelphia, 46; Brooklyn, 19; St. Louis, 35; Boston, 33; Baltimore, 41; San Francisco, 35; Cincinnati, 46; Cleveland, 56; Buffalo, 35; New Orleans, 34; Pittsburg, 82; Washington, 73; Milwaukee, 27; Newark, 38; Minneapolis, 51; Jersey City, 62; Louisville, 72; Omaha, 20; Rochester, 26; St. Paul, 29; Providence, 34; Denver, 77.

Investigation shows that the percentage of deaths, as is to be expected, is in keeping with the purity of the water supply of the different cities, and Pittsburg occupies the unenviable position at the head of the list. The cause for this is not difficult to find. For many years this municipality has been in the power of a director of public works who openly denied the value of scientists' opinions and scientific methods of procedure with reference to the supply of drinking-water, and this in the face of the fact that typhoid fever was endemic and often epidemic in the towns and villages located on the Alleghany river above the influent pipes of the water-works. Is it surprising, therefore, that Pittsburg has contributed more victims to this preventable disease than any other city of its size in the country?

After long agitation, a Sanitation Committee was appointed by councils, which, after a full investigation, reported in favor of a sand filtration system as a remedy for the appalling death-rate. This recommendation, submitted to the voters of the city in September last, was adopted by a large majority, and the prediction may be made that in the near future the mortality statistics from typhoid fever will drop to near the bottom of the list of the cities named.

One of the principal arguments advanced by the director already referred to in favor of the potability of the water was, that the typhoid bacilli could not be found in samples of water drawn from the river. In combating this ignorant idea, Professor William T. Sedgwick, of the Massachusetts Institute of Technology, and consulting biologist to the Sanitation Commission, made use of the following most appropriate simile:

"The germs of typhoid fever bear, on the contrary, to the harmless germs of sewage some such relation as do murderers to ordinary citizens in the passing crowd upon the busy street. They are very few in number, and outwardly resemble very closely the law-abiding. Even expert detectives on the watch may not be able to discover them. It is only afterwards that, often unseen and unsuspected, they do their deadly work. Even then they may escape detection in the crowd, and it is only when circumstantial evidence is overwhelming, or when they are taken in the very act, red-handed, that their true character becomes manifest."

The conditions that permitted Pittsburg to yield this enormous annual death-rate from typhoid fever are to be found in the fact that the sanitary authorities were powerless to exert any influence over the department having control over the supply of drinking-water and the same conditions are doubtless operative in other cities with high mortality rates from typhoid fever.—*Penn. Med. Journal.*

---

### THE WOUNDED IN SOUTH AFRICA.

Reports are rapidly coming from the seat of war in South Africa which bear out the more limited experience of our late war with Spain regarding the effect of the modern rifle bullet, and particularly of the Mauser, which is now universally regarded as the most humane of those in use. Sir William MacCormac, writing to the *Lancet,* gives various interesting details regarding wounds inflicted by Mauser bullets, wounds which a few years ago would have been thought quite incompatible with life. Sir William says: "One cannot help contrasting with amazement the comparative harmlessness of the injuries so frequently inflicted with the Mauser rifle bullet with the frightful extent of the damage done by those of the needle-gun and the Chassepôt. To any one familiar with the wounds caused by these weapons, many of those inflicted by the Mauser rifle might be regarded as being somewhat of the character of a 'pin prick.' Quite a large proportion of the wounded, in fact, have returned to duty, and several patients whom I have seen have been wounded for the second time at another engagement."

Some of the cases recorded are as follows: A man was struck by a Mauser bullet an inch above the symphysis pubis, the exit wound being one inch above the level of the anus and somewhat to one side. After being wounded he ran about one-third of a mile without inconvenience, and at the end of a fortnight had completely recovered. A soldier was shot in such a way that the bullet passed through the stomach after having penetrated the ilium. The patient suffered no inconvenience whatever, and was able to eat as usual. A bullet wound through the skull with protrusion of brain substance, dressed on the field, gave rise to no symptoms, and the wounds were healed on the fourth day. Penetrating wounds of the lungs in several instances recorded were followed by a quick recovery.

These are some of the cases to which special allusion is made, and it is certainly not too much to say, as has elsewhere been suggested, that the surgical aspects of bullet wounds must be largely rewritten, in view of the present unfortunate rapidly accumulating experience.

On the other hand it would appear that the Mauser is not always so merciful as the above cases would seem to indicate. Surgeon C. Marsh Beadnell, in a letter to the *British Medical Journal,* speaks for example, of a poor fellow "raving mad, who had been wandering about for hours with a portion of his frontal lobe protruding through a Mauser exit wound in the fore part of his skull." Another case of considerable interest reported by the same writer, relative to the effect of the shock of an exploding shell, is as follows: "A shell exploded about ten yards over a Highlander's head; he was untouched by any fragments, but the concussion must have produced some curious pathological change in his nervous system, as he has never ceased (now ten hours) swaying his head to and fro with a pendulum-like motion similar to that of the china dolls with the nodding heads so commonly seen in the London streets. His intellectual faculties have also been considerably disturbed, as he is only half rational." A collection of such cases bearing upon the effects of battle, mechanical or otherwise, would be of great value toward determining certain difficult points in connection with the results of trauma upon the nervous system, as we have previously had occasion to suggest in these columns. In general, we may certainly look for a large collection of data from medical men in the field which will prove of lasting scientific value; a poor compensation, but still a slight one, for the wholesale suffering which such a war of necessity entails.

Dr. Beadnell considers from his investigations on the effect of bullet wounds that those wounds which heal up most rapidly and give the least trouble are wounds produced by bullets having these characteristics:

(1) A very high velocity.

(2) A flat trajectory, so that they hit apex first and do not keyhole.

(3) A hard smooth sheath with a smooth rounded apex.

(4) A close range, for the same reason as (2) a bullet at a long range may hit the object side on.

He says: "If I were to tabulate these missiles in the order in which the wounds produced by them were progressively more serious from above downwards, the table would read something as follows:

"(1) Mauser.

"(2) Krag-Jorgensen.

"(3) Lee-Metford.

"(4) Man-stopping Lee-Metford (hollow-headed nickel-sheathed).

"(5) Any of the first three with the nickel sheaths around the apex removed so that the lead nucleus is exposed.

"(6) Dum-dum.

"(7) Remington brass-coated, as used by the Filipinos.

"(8) Remington lead bullets or the Martini-Henry.

"(9) Remington brass bullet, with brass sheathing removed so as to expose the lead nucleus.

"(10) Shrapnel bullets.

"(11) Shell or their fragments.

"I have no acquaintance with explosive bullets: it is said the Boers have used them, but I think this to be exceedingly doubtful. Most thoughtful men will agree that the Boers have proved themselves to be brave men, and instances of a violation of the laws of civilized warfare are few and far between."

In general, the health of the troops appears to have been excellent; diarrhea, sunstroke and sunburn have been the most annoying maladies.

With regard to the latter trouble the report continues: "The Highlanders are chiefly afflicted; why are men sent out in this fierce sun in petticoats? It is all very well to talk about the uniform being the essence of *esprit de corps*, but is not this carrying it too far? Surely they might be allowed to go into action at any rate as other men go. As it is their legs become covered with crops of vesicles; few of the men like their dress for this kind of work; the beauty of the kilt and sporran is masked by a khaki flap, and when these two part company the aspect is both ludicrous and alarming; I saw one man returning yesterday from his brush with the enemy in a pitiable plight; in his own words, he had had to 'take' a barbed wire entanglement, 'at the double,' and emerged 'a bleeding mass, with kilt hard a starboard, his khaki flap half-left turn, and his sporran dangling on the wire.'"—*Boston Medical and Surgical Journal.*

---

### Abdominal Exploration.

A better title for Dr. Howard A. Kelley's paper on exploration of the abdomen, in the *Medical News*, December 16, 1899, would have been Acts 17:27: "If happily they might feel after him and find him," since "The exploration of the abdomen as an adjunct to every celiotomy" is a rather surprising title. Dr. Kelley's experience at post-mortems in removing the thoracic organs through the vagina might suggest a new method for the removal of effusions from the pleural cavity and for the extraction of bullets from the thorax, since the *constrictor vaginæ* muscle might, by closure upon the operator's arm, make the operation anaerobic and thus decrease the possibility of sepsis. To be sure Dr. Kelley says that in cases in which "the immediate neighborhood of the incision is so septic as to demand the use of drainage, the operator would best forego the advantage of more extended examination." Since, however, advancing surgical technic constantly decreases the number of cases in which drainage is employed, the tendency would be to apply the method of exploration to a correspondingly large number of cases.

To explore the entire abdomen in special cases is not a new suggestion. Years ago J. Knowsley Thornton advocated abdominal nephrectomy, since the condition of each kidney could thus be ascertained before extirpation was undertaken. No skilled surgeon hesitates in special cases to explore any part of the abdomen. To adopt this, however, as a routine method may possibly seem to some surgeons extreme. Medical men who treat patients for various independent diseases at the same time, might usually, if more skilled diagnosticians, trace what are complications of a single disease back to the original cause. If Dr. Kelley's method were adopted, what would become of the favorite method of performing oophorectomy through a two-inch incision? How could the operator avail himself of the gridiron method of performing appendectomy after the manner of McBurney? Might it not relegate into innocuous desuetude appendectomy with "an inch-and-a-half incision and a week-and-a-half in bed"? Perish the thought! Rather than to recommend the thought as general, would it not be wiser to point out those conditions under which it is advisable, limiting it somewhat according to the indications of the individual case? When removing the tubes and ovaries it is not far to remove the appendix, only were gynecologist to be followed in their wide advocacy of the former operation and were their field of activity to be extended to the appendix in all cases, as some of them now recommend, must not the method be looked upon with grave apprehension by the general surgeon, as depriving him of his legitimate livelihood? When it comes from trachelorrhaphy to invading ruthlessly the privacy of the entire abdominal cavity, the general surgeon can but exclaim, "Behold, how great a matter a little fire kindleth." To what limits may a "touch course" in gynecology extend!! Really one can contemplate with a considerable degree of complacency an examination of a second kidney when an operation upon the other is under consideration, and in cases of uncertainty, as between gall-stones and renal calculi the doubt may well be settled. In cases of necessity the surgeon may safely pass through his fingers the whole intestine from the duodenum to the rectum, but deliberately in all cases, when by chance the belly is opened, to handle every organ, even if it be "with gloves," is perhaps further than the surgeon would be willing to follow the gynecologist. The method might in rare cases lead to some unexpected discovery. One is reminded of the two Jew friends who separated, one to the North and the other to the South, in exploiting a "Safe kidney and liver cure." Meeting some months later in New York, Jacob said to his colleague, "Isaac, I have done a great business, I have wonderful testimonials, which I shall read to you. Listen: 'I have for three months past taken weekly two bottles of Dr. Solomon's Safe Kidney and Liver Cure, and whereas I was on the verge of the grave I have gained fifty pounds and am now completely restored to health.'" "That is nothing," replied Isaac, "listen to my testimonials: 'I was born without liver and without lights, and was almost dead. I have taken ten bottles of Mr. Solomon's Safe Kidney and Liver Cure and now have a ten-pound liver and electric lights.'"

When a disease in one organ may be complicated by an extension of the process to others, when there are symptoms or conditions which suggest the possibility of independent disease elsewhere, it is doubtless desirable and has long been the practice of surgeons to utilize an opening in the abdominal cavity to explore and settle as far as possible all questions of diagnosis, and also to remove diseased tissues. It is a different matter to recommend the method as universal.

When a patient is in a feeble condition, little able to endure prolonged operation or manipulation, when the diagnosis of a given disease is fairly clear, and when there is absolutely nothing pointing to the existence of independent affections elsewhere in the abdomen, may not one confide in the kindliness of nature and that conservative experience which commonly finds the primary cause of disease single rather than multiple? May we not adopt the familiar words of Cardinal Newman when he sings: "I do not ask to see the distant scene, one step enough for me?"—*Cleveland Journal of Medicine.*

### PROGRESS IN THERAPEUTICS.

Whether we have reached the end of an old century or the beginning of a new one, a retrospective glance at last year's progress in therapeutics must satisfy us that a commendable amount of zeal and mental activity has been displayed in this branch of medical science, not so much perhaps in the matter of discovering new methods of treatment as in the systematization and organization of knowledge already acquired.

This broadening of common knowledge has been well exemplified in the matter of tuberculosis, where great progress has been made in grasping the significance of prophylactic treatment. The establishment of institutions for the isolation and treatment of tuberculosis has not as yet been put into general effect, but the indications are that this will very speedily take place. The destruction of sputum is very generally carried out. In the treatment of bronchopulmonary affections probably the most notable contribution of the year has been that of Jakob and others, showing the extreme value of carbonate of creosote in the initial stages of lung disease. Given in full doses of forty grains four times a day, it is said to exhibit an immediate ameliorating effect.

Inoculative methods of treatment have been tried principally in regard to the plague. The results of this method of treatment have not as yet been fairly estimated, but, in the opinion of many observers, a mortality saving of forty per cent. may be considered as resulting from a serum treatment of this disease. The preventive treatment of typhoid fever by means of antityphoid serum is also a matter upon which a positive verdict must be suspended. The opportunities for exhibiting this treatment at present existing in South Africa will probably result in some definite increase in our knowledge.

Since the establishment beyond dispute of the curative and preventive virtues of antitoxin in diphtheria, serumtherapy has scored no more signal triumph than that which it has this year achieved in the treatment of snake-bite by antivenene serum. Evidence is daily accumulating that if given within three hours after snake-bite, antivenene serum is capable of saving life. Anti-streptococcic serum has been used very largely during the past year, and a preponderance of evidence exists of its undoubted value in cases of septicemia of various kinds.

Medical literature abounds in cases demonstrating the great value of intravenous injections of normal saline solution in various toxemic conditions. This is likely to become an ordinary therapeutic expedient in the treatment of diabetic coma, uremic conditions, and other toxemias. A notable contribution to therapeutics has been the device of Finsen, of Copenhagen, whereby he treats cases of lupus vulgaris by the concentrated chemical rays of light. He reports 350 patients having been subjected to this method of treatment, and in nearly all cases the disease was arrested and the cosmetic results excellent. Schiff, of Vienna, has used the X-rays for a similar purpose. A remarkable success has also been found to accompany the X-rays for the removal of superfluous hairs.

Among the many valuable therapeutical suggestions of the year may be mentioned the use of methylene blue in diabetes, erythrol tetranitrate in arterial tension, suprarenal extract in asthma, urotropin in cystitis and uricemia, bromalin in epilepsy, and formaldehyde in hyperidrosis.—*Medical Age.*

---

### THE FRENCH USE THEM.

"The French are high livers. Their concessions to the palate are often great burdens to the stomach—banquets, midnight meals, wines, cordials and liqueurs too frequently and generously indulged upset the digestion, perturb or benumb the glandular system of the entire alimentary tract. But the Frenchman never thinks to stop so long as he can get relief from drugs. 'Antikamnia Laxative Tablets' are said to be extensively used, aiding him to fill a want he has perhaps too often felt. These tablets not only relieve the disagreeable 'heaviness' after a gluttonous meal, but they act as stomachic-tonic, gently stimulate the intestinal peristalsis, facilitate the glandular functions of secretion and excretion, allay fermentative processes, restore equilibrium to the nervous centers, hasten the elimination of alcoholic by-products and prevent the usual gastralgic symptoms."—DR. L. P. HAMMOND.

